# Degradation of Toluene from Gas Streams by Heterogeneous Fenton Oxidation in a Slurry Bubble Reactor with Activated Carbon-Based Catalysts

**DOI:** 10.3390/nano12193274

**Published:** 2022-09-21

**Authors:** Emanuel F. S. Sampaio, V. Guimarães, O. S. G. P. Soares, M. Fernando R. Pereira, Carmen S. D. Rodrigues, Luis M. Madeira

**Affiliations:** 1 LEPABE—Laboratory for Process Engineering, Environment, Biotechnology and Energy, Faculty of Engineering, University of Porto, Rua Dr. Roberto Frias, 4200-465 Porto, Portugal; 2 LSRE–LCM—Laboratory of Separation and Reaction Engineering—Laboratory of Catalysis and Materials, Faculty of Engineering, University of Porto, Rua Dr. Roberto Frias, 4200-465 Porto, Portugal; 3 ALiCE—Associated Laboratory in Chemical Engineering, Faculty of Engineering, University of Porto, Rua Dr. Roberto Frias, 4200-465 Porto, Portugal

**Keywords:** heterogeneous Fenton oxidation, volatile organic compounds, toluene, bubble reactor, activated carbon

## Abstract

A novel approach for the treatment of volatile organic compounds from gaseous streams was developed. In order to accomplish this, a semi-batch bubble reactor was used, aiming to assess the toluene (selected as model compound) degradation from gaseous streams via heterogeneous Fenton oxidation. Activated carbon-based catalysts—metal-free or iron-impregnated—with different textural and chemical surface properties were used for the first time as catalysts, in order to degrade gaseous toluene using such technology. Complementary characterization techniques, such as nitrogen adsorption at –196 °C, elemental analysis, pH at the point of zero charge (pH_PZC_), inductively coupled plasma optical emission spectrometry (ICP-OES) and transmission electron microscopy (TEM), were used. The materials’ chemical surface properties, particularly the presence of N-surface groups, were herein found to play an important role in toluene adsorption and catalytic performance. The maximum amount of toluene transferred, 6.39 × 10^−3^ mol, was achieved using melamine-doped activated carbon (N-doped material) that was impregnated with iron (sample herein called ACM-Fe). This iron-based catalyst was found to be quite stable during three reutilization cycles.

## 1. Introduction

The emissions of volatile organic compounds (VOCs), such as benzene, toluene, ethylbenzene and xylene (BTEX), from chemical and petrochemical industries into the atmosphere, are responsible for adverse effects on the environment and public health [1,2,3]. VOCs act as a precursor for the formation of photochemical smog, secondary organic aerosol and ozone. Many VOCs are carcinogenic and toxic, and when inhaled or ingested, can also cause mutagenic and teratogenic effects [4,5,6,7]. The BTEX compounds exhibit high toxicity levels, for instance toluene [8], and are among the 129 priority pollutants listed by the U.S. Environmental Protection Agency [9]. Thus, it is crucial to remove them from gas-polluted streams, or at least reduce their concentrations to levels that meet gaseous emissions legislation standards.

Some technologies have been developed for gaseous toluene removal, including absorption [10], adsorption [11,12,13] and biofiltration [14]; however, these technologies only transfer the pollutant from one phase to another, and do not provide effective degradation; thus, subsequent treatments are often necessary. As a result, destructive processes are considered to be more attractive.

Advanced oxidation processes (AOPs) have emerged as destructive techniques, and interest in them has increased significantly in recent years [15]. AOPs are based on the generation of highly reactive and non-selective species, namely hydroxyl radicals (HO^•^) [16], which have an oxidation potential of 2.8 V [17]. Such species are capable of oxidizing organic (and also inorganic) pollutants into smaller compounds (intermediate species), and ultimately into carbon dioxide, water and inorganic ions [18]. Among AOPs, Fenton’s reaction is a classic AOP, and one of the most studied technologies in wastewater treatment. In this process, the generation of HO^•^ radicals occurs through the catalytic action of ferrous ions (Fe^2+^) to decompose hydrogen peroxide (H_2_O_2_) in an acidic environment (Equation (1)) [19,20]. On the other hand, catalyst regeneration results, among other things, from the reaction between H_2_O_2_ and Fe^3+^ (Equation (2)), yielding hydroperoxyl radicals with an oxidation potential of 1.7 V [21], making them less reactive than HO^•^. Finally, Equations (3) and (4) show organic species oxidation by the HO^•^ species that are produced in liquid medium, yielding oxidized products (i.e., intermediate compounds) which can be completely oxidized into CO_2_ and H_2_O.

(1) $${\mathrm{Fe}}^{2+}{+H}_{2}O_{2\;}\to {\;\mathrm{Fe}}^{3+}{+\mathrm{HO}}^{•}{+\mathrm{OH}}^{-}$$

(2) $${\mathrm{Fe}}^{3+}{+H}_{2}O_{2}\;\to {\;\mathrm{Fe}}^{2+}{+\mathrm{HOO}}^{•}{+H}^{+}$$

(3) $${\mathrm{HO}}^{•}+\mathrm{Toluene}\;\to \;\mathrm{Oxidation}\;\mathrm{Products}\;$$

(4) $${\mathrm{HO}}^{•}+\mathrm{Oxidation}\;\mathrm{Products}\;\to \;{\mathrm{CO}}_{2}+H_{2}O$$

Fenton’s reaction has some advantages, such as a low investment cost, high efficiency and ease of application under smooth operational conditions (atmospheric pressure and room temperature). It is considered a “green technology”, since it uses environmentally friendly reagents [22]. However, the homogeneous process presents drawbacks that are related to the use of a high concentration of iron ions (commonly in the 50–80 mg dm^−3^ range) and/or the necessity of their removal/recovery at the end of the process in order to comply with legal standards; this typically implies a subsequent (i.e., downstream) treatment unit. The heterogeneous Fenton-like process can overcome these drawbacks through the use of heterogeneous catalysts, i.e., with iron or other transition metal species supported on a porous solid matrix [23,24]. 

The heterogeneous Fenton-like process presents the same principles as the homogeneous system, albeit in a slightly more complex manner than the latter due to competing phenomena such as adsorption and reaction. In a very simplified way, the process can be described with two main reactions, shown in Equations (5) and (6), which illustrate hydroxyl radical production and ferrous iron regeneration [24]:

(5) $${X-\mathrm{Fe}}^{2+}{+H}_{2}O_{2\;}\to {\;X-\mathrm{Fe}}^{3+}{+\mathrm{HO}}^{•}{+\mathrm{OH}}^{-}$$

(6) $${X-\mathrm{Fe}}^{3+}{+H}_{2}O_{2}\;\to {\;X-\mathrm{Fe}}^{2+}{+\mathrm{HOO}}^{•}{+H}^{+}$$

where X represents the solid support.

**Figure 1:**
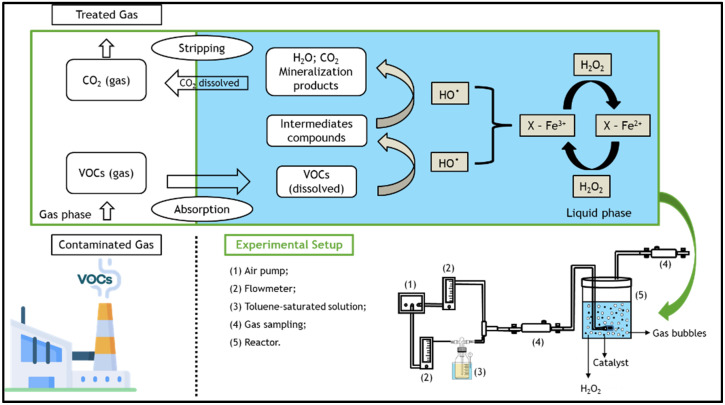
Schematic of the main processes involved in the degradation of volatile organic compounds (VOCs) present in the gaseous streams through a bubble reactor by the heterogeneous Fenton process, as well as details of the setup used in all experiments.

However, in order to treat organic compounds in gas streams using heterogeneous (or homogeneous) Fenton-like processes, it is necessary to transfer the pollutants from the gaseous to the liquid phase, wherein the Fenton reagents are present [25,26]. The overall process consists of several steps: first, gaseous toluene is transferred to the liquid phase by absorption [1], where it is then oxidized by the hydroxyl radicals generated by Fenton’s reaction [27]—see Figure 1. The degradation of dissolved toluene increases the driving force for more toluene to be transferred from the gas to the liquid. Finally, the clean gaseous phase is separated from the aqueous phase [28]. However, some VOCs are hydrophobic compounds that have low water solubilities (e.g., solubility of toluene in water = 5.71 × 10^−3^ mol dm^−3^ at 25 °C [29]); thus, the application of activated carbon-based heterogeneous catalysts appear to be promising, as they significantly increase the gas-liquid transfer of VOCs [30] through adsorption. In this manner, one can also take advantage of heterogeneous Fenton oxidation in the liquid–solid system. 

This research studied the catalytic oxidation of gaseous toluene via the heterogeneous Fenton-like process using activated carbons (ACs) with distinct surface chemical properties; the ACs were used as catalysts by themselves (as metal-free catalysts), or as supports of iron-based catalysts. Carbon materials have been widely employed as catalysts/adsorbents, namely for application in AOPs, and they have been demonstrated to possess surface chemical properties that influence oxidation performance [24,31,32]. Among carbon materials, ACs are particularly attractive as a result of their developed porosities and high surface areas that are stable across a wide range of pH values, as well as for their tunable surface chemistry [33]. Rodrigues et al. [24] assessed the degradation of *p*-nitrophenol by wet peroxidation and heterogeneous Fenton oxidation over activated carbon-based catalysts, and found that their chemical surface properties play an important role in catalytic performance. Activated carbons with oxygenated surface groups presented the worst performance, contrary to N-doped ACs. In the latter case, nitrogen groups increase the π electronic density, which improves interactions between the carbon surface and the pollutant. Nevertheless, AC-based iron catalysts are the most reported carbon-derived materials that are used for heterogeneous Fenton-like oxidation of organic compounds [24,34,35,36,37]. To the best of the authors’ knowledge, no studies in the open literature have yet used iron supported in ACs in a heterogeneous Fenton reaction for the degradation of organic pollutants present in gaseous streams.

In the present study, a commercial activated carbon (Norit GAC 1240 AF) was modified by chemical and thermal treatments to obtain materials with distinct surface chemistries, while maintaining the original textural features. Posteriorly, the activity levels of the modified activated carbons and iron-derived catalysts (obtained upon impregnation of the carbon materials) were assessed on the degradation of toluene from gas streams using a semi-batch bubble reactor. The iron-based catalyst that provided the best catalytic performance was then used in successive reutilization cycles to evaluate its stability.

## 2. Materials and Methods

### 2.1. Materials

Hydrogen peroxide (H_2_O_2_, 30% *w/v*), ferrous sulfate heptahydrate (FeSO_4_.7H_2_O, 99.0% purity), sodium sulfite (Na_2_SO_3_, 98.0% purity), sodium hydroxide (NaOH, 99.0% purity), sulfuric acid (H_2_SO_4_, 97–98% purity) and nitric acid (HNO_3_, 65% purity) were purchased from Panreac. Pure liquid toluene (99% purity) (chemical formula C_7_H_8_, molar mass = 92.14 g mol^−1^) and di-sodium hydrogen phosphate anhydrous (99% purity) were purchased from VWR Chemicals.

A commercial activated carbon, Norit GAC 1240 AF, was used as the initial material to prepare all modified activated carbons and AC-Fe catalysts. Melamine (C_3_H_6_N_6_, ≥ 99% purity) and iron (III) nitrate nonahydrate (FeH_18_N_3_O_18_, 99% purity) were obtained from Fluka.

### 2.2. Activated Carbons Functionalization

Initially, the Norit activated carbon was ground and sieved to obtain a particle diameter of 0.1–0.3 mm. These particles, labelled as AC_0_, were used as starting material. Then, sample AC_0_ was modified using chemical and thermal treatments to produce materials with different chemical and textural properties; the nomenclature and treatments applied are summarized in Table 1.

The sample AC_0_ (9 g) was oxidized using acid treatment with HNO_3_ in a 125-milliliter Soxhlet extraction apparatus that was connected to a boiling flask and a condenser. In order to accomplish this, a volume of 0.300 dm^3^ of 6 mol dm^−3^ HNO_3_ was introduced into a 0.5 dm^3^ round-bottom flask and heated for 3 h until the boiling temperature was reached, using a heating mantle. After that, the oxidized sample was thoroughly washed with distilled water to reach a neutral pH, and dried at 110 °C overnight. This sample is called ACH.

Posteriorly, ACH was used to prepare the samples via gas-phase thermal treatments. Using thermal treatments at different temperatures allows one to selectively remove the oxygen groups that are present on the surface of ACH [24,31,38]. Thus, the ACH sample was heat treated at 400, 600 and 900 °C for 1 h under nitrogen flow, yielding the samples herein called ACH_400_, ACH_600_ and ACH_900_, respectively—cf. Table 1.

Lastly, N-doped sample (called ACM) was also obtained from AC_0_ using melamine as a nitrogen precursor. In order to accomplish this, 0.6 g of the AC_0_ sample and 0.4 g of melamine were dipped into 0.050 dm^3^ of a 1 mol dm^−3^ aqueous solution of ethanol (96% *v*/*v*), and stirred at room temperature for 24 h. Afterwards, the sample was filtered, washed with distilled water and dried at 100 °C overnight. Posteriorly, the solid was heat treated at 600 °C for 1 h under nitrogen flow in order to obtain the ACM sample.

### 2.3. Catalysts Preparation

The iron-based catalysts were prepared using the incipient wetness impregnation method of the different modified activated carbons described in the previous section. The impregnation was carried out under vacuum and ultrasonic mixing, using iron (III) nitrate nonahydrate as a precursor solution to obtain an iron content of 2 wt.%. Thereafter, samples were dried at 100 °C overnight, heat treated under N_2_ flow for 1 h, and finally reduced under H_2_ flow for 3 h at 400 °C. The samples are denominated ACXy-Fe. The reduction step reduces Fe^3+^ to Fe^2+^, since the latter species is necessary to enhance Fenton process performance; the reason for this is because the kinetic constant for Equation (1) is significantly higher than the one for iron regeneration—Equation (2). Moreover, the heat treatment applied also results in more stable materials, ensuring that minimal iron is leached out during the catalytic experiments.

### 2.4. Carbon Materials Characterization

The modified activated carbons and their respective catalysts were characterized with N_2_ adsorption/desorption isotherms at –196 °C, elemental analysis, pH at the point of zero charge (pH_PZC_), transmission electron microscopy (TEM) and inductively coupled plasma optical emission spectrometry (ICP-OES).

The textural characterization of the samples was assessed with N_2_ adsorption isotherms, determined at −196 °C with a Quantachrome NOVA 4200e apparatus. The samples (∼0.1 g) were outgassed at 150 °C for 3 h under vacuum. The surface area (*S*_BET_) was determined using the Brunauer–Emmett–Teller (BET) equation [39,40]. The mesopore surface area (*S*_meso_) and the micropore volume (*V*_micro_) were calculated using the *t*-method [40,41]. The total specific pore volume (*V*_p_) was determined from the amount of N_2_ adsorbed at *P*/*P*_0_ = 0.95 [40]. The pore size distribution was determined by applying the DFT method to the N_2_ adsorption isotherms [40].

The amounts of carbon, hydrogen, nitrogen and sulphur were determined with elemental analysis using a Vario micro cube analyzer (Elementar GmbH), by combusting the sample at 1050 °C. The oxygen content was determined using a rapid oxy cube analyzer (Elementar GmbH) in which the sample underwent pyrolysis at 1450 °C. Each sample was analyzed in triplicate. The pH at the point of zero charge (pH_PZC_) was determined by mixing 0.025 g of each carbon material with 0.025 dm^3^ of a NaCl solution (0.01 mol dm^−3^). The pH was adjusted using HCl or NaOH solution (0.01 mol dm^−3^) to obtain values between 2 and 10. The final pH was measured after 24 h under stirring at room temperature. The pH_PZC_ value of each carbon material was determined when the pH_final_ vs. pH_initial_ curve crossed the line pH_final_ = pH_initial_ [42].

Thermogravimetric analysis (TGA) was performed to confirm the effect of the thermal treatments (i.e., functionalization) on the modified activated carbons, and to complement the elemental analysis results. It was carried out using an STA 409 PC/4/H Luxx Netzsch, by heating the samples under N_2_ flow from 50 to 900 °C, at a heating rate of 10 °C min^−1^, and posteriorly, two isothermal steps were performed: 7 min under N_2_ and 13 min under air, both at 900 °C.

Transmission electron microscopy (TEM) analyses were carried out in a Jeol JEM 1400 (Zeiss, model EM 10C) electron microscope equipped with a SC1000 Orius™ CCD camera operated at 80 kV; the sample was dispersed in ethanol before analysis. The metallic dispersion (*D*_M_) of the iron particles was calculated according to Equation (7) [40], assuming spherical metal particles:

(7) $$D_{M}=\frac{6\times n_{S}\times M\times 1000\;}{\rho \times N\times d_{p}}\times 100$$

where $n_{S}$ is the number of atoms at the surface per unit area (1.63 × 10^19^ m^−2^ for Fe [40]), *M* is the molar mass of iron (55.84 g mol^−1^), ρ is the density of iron (7.87 g cm^−3^), *N* is Avogadro’s number (6.022 × 10^23^ mol^−1^) and $d_{p}$ is the average iron particle size (nm) [40].

The iron content of the catalysts was assessed by ICP-OES using an iCAP 7000 spectrophotometer from Thermo Scientific (Waltham, MA, USA). Prior to the analysis, the materials were dissolved in a mixture of 0.007 dm^3^ of HNO_3_ 65% (*v*/*v*) and 0.001 dm^3^ of H_2_O_2_ 30% (*v*/*v*), using a Start D Microwave Digestion System from Milestone (Sorisole, Italy).

### 2.5. Experimental Procedure

#### 2.5.1. Simulated Toluene Gas Stream

The polluted gas stream, i.e., the simulated toluene gas stream, was produced by the stripping of toluene according to a method described elsewhere [25,43]. In brief, a clean air stream was bubbled into a 0.5 dm^3^ washing bottle (Duran) containing ~0.3 dm^3^ of pure liquid toluene. The stripping of toluene was realized at 5 °C (using a Polystat CC1 thermostatic bath from Huber) and atmospheric pressure. The air stream was supplied continuously at a flow rate (*Q*_air_) of 0.05 dm^3^ min^−1^. Then, the concentrated toluene stream was taken to a mixing bottle, where a new clean air stream was fed at a flow rate of 0.1 dm^3^ min^−1^ in order to dilute the toluene-containing stream. The diluted gas stream was then fed to the reactor. All gas flow rates were measured at room temperature and atmospheric pressure. Gas flow rates were measured using two direct-reading flow meters (Cole Parmer) coupled with non-return valves (Swagelok, model 1/3 psi). The experimental setup is shown in Figure 1, along with a schematic of the processes involved.

#### 2.5.2. Adsorption and Reaction Experiments

The experiments were performed in a semi-batch bubble reactor (BR), equipped with an internal cylindrical air diffuser (inert stone, *H* = 2.5 cm and *D* = 1.5 cm) to form the gas bubbles in the liquid medium, thereby allowing the mass transfer of the simulated toluene gas stream for the distilled water (cf. detail in Figure 1). In each experiment, 0.300 dm^3^ of distilled water with an adjusted pH ~ 3.0 (using 1 mol dm^−3^ H_2_SO_4_) was added into the reactor. Then, the modified activated carbon/catalyst (ACX_y_/ACX_y_-Fe) was added, and the diluted gas stream began to bubble in the reactor. This moment was considered time zero for the adsorption experiments. In wet peroxidation and Fenton’s oxidation experiments (i.e., when using the metal-free ACs or the AC-Fe catalysts, respectively), the desired amount of H_2_O_2_ was added to the reactor immediately before bubbling the gas stream. It is noteworthy that no stirring was required, as the applied gas flow rate created enough turbulence to keep the carbon materials in suspension in the liquid phase (thus operating the bubble reactor as a semi-batch slurry device).

Periodically, gas samples were taken to measure the toluene concentration in the gaseous phase using gas chromatography at both reactor inlet and outlet streams. Still, during the catalytic oxidation experiments, samples (0.005 dm^3^) were taken along time in order to measure the dissolved organic carbon (DOC) concentration and the residual hydrogen peroxide concentration in the liquid phase. Before the analyses, such samples were filtered to remove the metal-free catalyst or iron-based catalyst. The DOC concentration was assessed after stopping the homogeneous reaction catalysed by any leached iron, by adding excess sodium sulphite (which reacts instantaneously with residual hydrogen peroxide [44]).

The amount of toluene transferred from the gaseous to the liquid phase during the experiments (*η*—mol dm^−3^) was calculated through Equation (8):

(8) $$\eta =\frac{Q_{gas}\int_{0}^{\;t_{op}}\left(C_{in}-C_{out}\left(t\right)\right)dt}{M_{toluene}}$$

where *C_in_* = 0.017 g dm^−3^ and *C_out_* represent the gas inlet and outlet toluene concentrations (g dm^−3^), respectively; *Q_gas_* represents the gas feed flow rate (dm^3^ min^−1^); *t* is the time and *t_op_* the overall operation time (both in min); *M_toluene_* represents the molecular weight of toluene (g mol^−1^); and *V_liquid_* stands for the liquid phase volume inside the reactor (dm^3^).

### 2.6. Analytical Methods

The DOC was determined using catalytic combustion at 720 °C, according to method 5310 D [45], using a TOC-L analyser from Shimadzu.

Hydrogen peroxide in solution was determined following a colorimetric method as described by Sellers [46]. The yellow-orange colour of the complex formed from the reaction between hydrogen peroxide and titanium oxalate (IV) in the sample was measured at 400 nm, using a Heλios γ spectrophotometer (Thermo Electron Corporation).

Toluene concentrations in the gaseous phase were measured ex situ via gas chromatography (Agilent 7820A, column HP-5 M S) with flame ionization detection (GC-FID). The injector, detector and oven were operated at 220, 250 and 100 °C, respectively. Helium was used as the carrier gas, at a flow rate of 0.025 dm^3^ min^−1^. A sample of 100 µL of the gaseous phase was manually inserted into the injector, using a chromatography syringe (Hamilton).

All analytical methods were performed in duplicate, and the coefficient of variation was less than 2% for DOC, and 4% for the H_2_O_2_ concentration and toluene concentration determinations.

## 3. Results and Discussion

### 3.1. Carbon Materials Characterization

Table 2 summarizes the textural properties of the different modified activated carbons and iron-based catalysts, while the corresponding adsorption isotherms and pore size distributions are presented in Figure S1 in the Supporting Information section. The commercial material, sample AC_0_, presents an *S*_BET_ of 1047 m^2^ g^−1^, whereas the ACH (968 m^2^ g^−1^) sample shows a decrease in the surface area as a result of the acid treatment applied to AC_0_. The nitric acid treatment introduced a large amount of oxygen surface groups, which partially blocked the accessibility of N_2_ molecules to the smallest pores; this decreased the surface area of the oxidized sample [31,47]. On the other hand, the successive heat treatments that were applied at different temperatures (400, 600 and 900 °C) to the oxidized sample (ACH) selectively removed some oxygen surface groups, thereby increasing the BET specific surface area. The slight differences in the surface areas between treated samples can be explained by analytical errors, since for these materials, the equipment presented an error of ± 20 m^2^ g^−1^. The N-doped sample, ACM, presented the lowest BET surface area (807 m^2^ g^−1^) due to the large amount of nitrogen surface groups present on the surface that could partially block the access of N_2_ molecules within the small pores. The pore size distributions obtained by the DFT method were very similar for all samples, as seen in Figure S1c,d.

The iron-based catalysts did not present significant differences in their surface areas and total pore volumes, as compared to their modified activated carbons. However, the impregnation method incorporated iron species in the pores, or even in the small pores, and therefore partially blocked the access of N_2_ molecules inside them; this generally provoked a slight decrease in the BET surface areas. However, for the ACH-Fe sample, the BET surface area increased compared to its support; this result could be due to the thermal treatment at 400 °C that was applied after iron impregnation for Fe(III) reduction, which promoted the removal of some oxygen-containing surface groups.

Table 3 summarizes the carbon (C), hydrogen (H), nitrogen (N), sulphur (S) and oxygen (O) content and pH_PZC_ values of the modified activated carbons. The original sample, AC_0_, had an approximately neutral pH_PZC_ value, presenting only 5.3 wt.% of oxygen content. Sample ACH had the lowest pH_PZC_ value and the highest amount of oxygen (19.9 wt.%) due to oxidation with HNO_3_, which introduced a large amount of oxygen surface groups, including a large amount of carboxylic acids [38]. Therefore, the ACH sample presented acidic properties. Furthermore, the ACH sample exhibited a low nitrogen content (0.6 wt.%) that could be attributed to the formation of N-containing groups on the carbon surface during the oxidation with HNO_3_. The heat-treated samples contained a successively lower amount of oxygen, decreasing from 19.9 to 8.9 wt.% with increases in the applied heat treatment temperature, as verified in previous studies [24,31]. The heat-treated samples exhibited pH_PZC_ values corresponding to approximately neutral properties, meaning that the surface became more basic with increases in treatment temperature when compared to ACH. Finally, the N-doped sample, ACM, showed an approximately neutral to slightly basic pH_PZC_ value, and a nitrogen content of 7.0 wt.%. The total amount of elements reported in Table 3 is not 100 wt.% because, in addition to C, H, N, O and S, the prepared modified activated carbons also contained minor amounts of inorganic elements, which were not identified by our elemental analysis technique.

Table 4 summarizes the volatile content, fixed carbon and ash content of the modified activated carbons. Thermogravimetric analysis (TGA) demonstrated that (please refer to the TGA curves presented in Figure S2), under inert atmosphere, the modified activated carbons were quite stable. However, above a temperature of 100 °C, a decrease in the samples’ masses was observed, which related to the presence of oxygen or nitrogen surface groups. The volatile content was higher for ACH and ACM, once these samples are constituted by a large amount of oxygen and nitrogen groups, respectively; indeed, the sum of the nitrogen and oxygen content was the highest among all materials for those two samples (cf. Table 3). The volatile content of the heat-treated samples became successively lower with increases in heat treatment temperature, due to the selective removal of some oxygen surface groups. Upon changing the atmosphere to air, an abrupt decrease in the samples’ masses occurred due to burning of the AC, after which the masses of the samples remained constant until the end of the experiment; this was potentially linked to the metal content of the activated carbon (ash content), which did not burn.

Table 5 shows that the iron content was similar for all catalysts, and near the theoretical content (2 wt.%). Previous studies demonstrated that the iron content incorporated using the incipient wetness impregnation method over ACs is very similar to the theoretically determined content [24,48].

**Figure 2:**
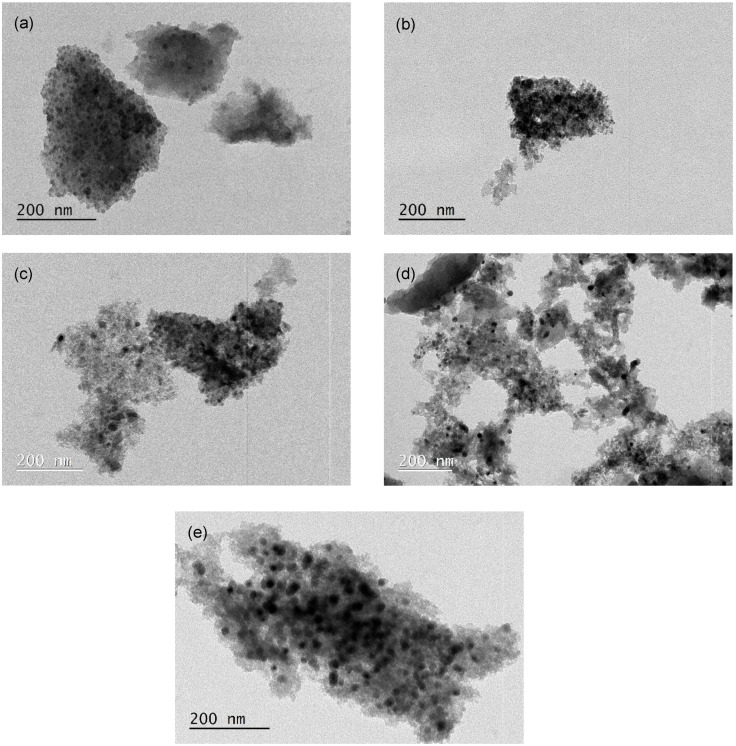
TEM images of the ACM-Fe fresh sample (**a**), the ACM-Fe used sample (**b**), the AC_0_-Fe fresh sample (**c**), the ACH-Fe fresh sample (**d**) and the ACH_900_-Fe fresh sample (**e**).

Figure 2 presents the TEM images for some catalysts used in this study. The fresh samples show a good iron dispersion (12.9%, 12.9%, 8.4% and 7.8%) with particle sizes in the range of 9.0 ± 3.0 nm, 9.0 ± 5.0 nm, 13.9 ± 3.1 nm and 15.0 ± 3.7 nm for ACM-Fe, AC_0_-Fe, ACH-Fe and ACH_900_-Fe samples, respectively. The particle size distribution of the samples is illustrated in Figure S3. Metal dispersion is expected to play an important role in Fenton’s reaction; however, in this study the samples exhibited similar values, and so no relationship was found between the metal dispersion and the samples’ performance. The particle diameter of the used ACM-Fe sample (12.5 ± 8.1 nm) was higher, while the metallic dispersion (9.3%) was lower than the fresh sample; this may be a consequence of the leaching phenomenon described below, particularly of the smaller loosely bound metallic particles, suggesting an increase in the particle diameter, and consequently a decrease in metallic dispersion.

### 3.2. Heterogeneous Fenton Reaction

#### 3.2.1. Processes Screening

**Figure 3:**
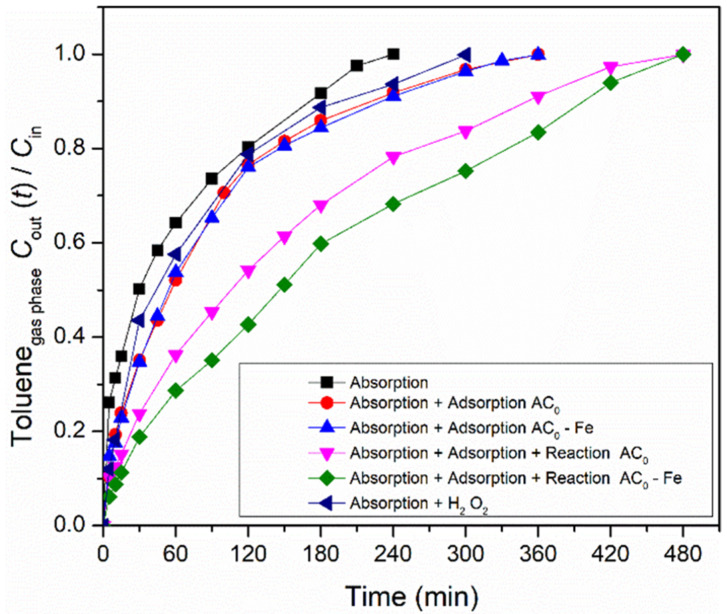
Screening of all processes (absorption, adsorption and reaction) that occurred during toluene removal/degradation by heterogeneous Fenton oxidation with AC_0_-related materials. Experimental conditions (when applicable/used) were as follows: pH_0_ = 3.0, [H_2_O_2_] = 0.005 mol dm^−3^, [modified activated carbon/iron-based catalyst] = 0.75 g dm^−3^, *Q*_air_ = 0.15 dm^3^ min^−1^.

The overall heterogeneous Fenton process is divided into three main contributions for toluene abatement from the gas stream: absorption of toluene in the liquid; adsorption over the carbon material; and reaction, catalyzed either by the modified activated carbon or by the iron-based catalyst. Thus, in order to find the contribution of each process, a processes screening was carried out in the semi-batch bubble reactor. The absorption experiment was performed without oxidant (hydrogen peroxide) and carbon material, while the modified activated carbon/iron-based catalysts were used in adsorption experiments (without any oxidant). Finally, the reaction experiments were conducted in the presence of oxidant and metal-free activated carbon/iron-based catalyst. Furthermore, a reaction using oxidant only also was performed. The operating conditions during the experiments were fixed in 5 mM of H_2_O_2_, 0.75 g dm^−3^ of carbon material, at pH = 3, T = 25 °C and Q_air_ = 0.15 dm^3^ min^−1^. The ratios between the toluene concentration in the gaseous phase at the outlet and inlet of the bubble reactor (*C_out_(t)/C_in_*) for all processes are shown in Figure 3, using the commercial activated carbon (AC_0_) as carbon material, and the corresponding Fe-impregnated catalyst.

Firstly, the adsorption experiment involving the mass transfer of toluene from the gaseous to the liquid phase was evaluated. The concentration of the outlet gaseous phase continuously increased for 240 min until the concentration became similar to the inlet phase (*C*_out_/*C*_in_ ~ 1.0). At this point, water saturation was reached (which corresponds to the solubility of toluene in water at 25 °C), thus allowing to transfer 1.66 × 10^−3^ mol of toluene. The experimental solubility obtained in this study was 5.54 × 10^−3^ mol dm^−3^ (cf. Equation (7)), which is equivalent to 0.51 g dm^−3^. Compared to the solubility reported by other authors at the same temperature (5.64 × 10^−3^ mol dm^−3^ at 25 °C [29]), our value is slightly lower, but the deviation is below 2%. The H_2_O_2_ per se permits the degradation of some toluene, since the reagent possesses oxidation properties (oxidation potential of 1.9 V [17]); this consequently increases the amount of toluene transferred (2.82 × 10^−3^ mol) compared to what is absorbed by water. 

As referred, the amount of toluene transferred by absorption is low. However, the amount of VOCs transferred increases with the use of activated carbons, due to their adsorption capacity [30]. Thus, adsorption experiments were carried out to improve the gas-liquid mass transfer. As expected, the experiments with samples AC_0_ and AC_0_-Fe were prolonged until 360 min, where the saturation point was reached, i.e., when the gaseous phase concentration at the outlet is equal to the inlet one. These results show the capacity of the carbon materials to adsorb toluene molecules as a result of their high specific surface area and surface properties. Overall, the adsorption processes (which also include absorption, because part of the toluene remained dissolved in water) allowed the toluene transfer of 3.17 × 10^−3^ mol and 3.18 × 10^−3^ mol, for samples AC_0_ and AC_0_-Fe, respectively, i.e., a nearly two-fold increase compared to the absorption run. In both adsorption experiments, a similar toluene transfer was obtained because the samples showed identical textural and presumably chemical properties (cf. Table 2). The increase in toluene transfer that was observed through adsorption experiments was also reported by Xie et al. [30] and Rodrigues et al. [49], where they evaluated the degradation of gaseous toluene using a zeolite (Fe/ZSM-5) and carbon-coated monoliths impregnated with iron (CM-Fe), respectively.

Furthermore, the catalytic activity of the metal-free catalyst and iron-based catalyst were also assessed under the same conditions as used in the adsorption runs, but with the addition of H_2_O_2_, the oxidant, to the liquid phase. As shown in Figure 3, the reaction experiments were further extended until the saturation point was reached (*t* = 480 min). When using the AC_0_-Fe catalyst, the highest amount of transferred toluene was reached, because the oxidation reaction that occurs in the liquid phase between the generated radicals and the toluene molecules increases the driving force to transfer more gas. This phenomenon was also observed with the AC_0_ sample, because it is well known that through wet peroxidation, ACs are able to catalyze the process [24,31]. However, the amount of toluene transferred, as calculated from Equation (7), was smaller compared with the heterogeneous Fenton oxidation using the AC_0_-Fe sample (4.80 × 10^−3^ mol vs. 5.17 × 10^−3^ mol).

The performances obtained by adsorption, wet peroxidation and heterogeneous Fenton oxidation support a conclusion that activated carbon-based materials are promising for the abatement of toluene from gas streams. In the following sections, the influences of the chemical and textural properties of the prepared activated carbon materials was evaluated for the adsorption and catalytic oxidation of gaseous toluene.

#### 3.2.2. Effects of the Materials’ Chemical and Textural Properties

**Figure 4:**
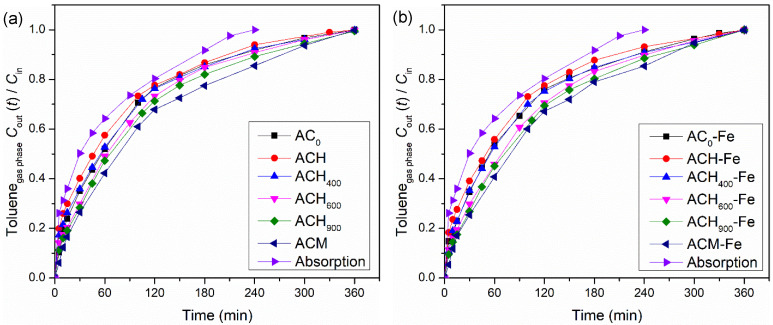
Toluene concentration at the outlet of the bubble reactor during absorption + adsorption experiments with modified activated carbon (**a**) and with iron-based catalysts (**b**). Experimental conditions were as follows: pH_0_ = 3.0, [modified activated carbon/iron-based catalyst] = 0.75 g dm^−3^, *Q*_air_ = 0.15 dm^3^ min^−1^.

Modified activated carbons were initially used in adsorption experiments to determine the effect of their surface chemistry and texture on toluene removal. These materials exhibit high BET surface areas (807–1072 m^2^ g^−1^—cf. Table 1); therefore, the amount of gaseous toluene that was transferred to the liquid phase was higher than in the simple absorption experiment, as shown in Figure 4a. Among all adsorption experiments, the ACH sample showed the lowest adsorption capacity, represented by the lowest amount of toluene transferred in the process (2.81 × 10^−3^ mol), which must be related to the high amount of oxygen surface groups and the lowest pH_PZC_ of this sample—Table 3. Moreover, the adsorption capacity of the heat-treated ACs increased upon increasing the temperature of the thermal treatment, since the oxygen-containing surface groups were successively removed (from ACH to ACH_900_)—Table 3. This behavior can be related to the fact that the carbon surface was negatively charged in sample ACH, with a pH_PZC_ lower than the solution pH (pH = 3); hence, the anionic toluene present in the liquid phase would be subjected to repulsion forces when it approached the surface of the material [24,31]. Moreover, the adsorption capacities of the heat-treated carbons were higher than the adsorption capacity of the ACH sample, due to the existence of a lower amount of oxygen surface groups, which favored electronic interactions between the carbon surface and the toluene molecules (due to the predominance of π electrons in the basal plane of carbon when there are fewer oxygenated surface groups) [24,50]. Among the heat-treated activated carbons, the highest toluene transfer was observed for the ACH_900_ sample (3.84 × 10^−3^ mol). However, the activated carbon that was doped with nitrogen, using melamine as precursor (ACM), achieved the best performance (η=4.24 × 10^−3^ mol). The presence of N-containing groups on the carbon surface increased the electronic density, favoring interactions between the toluene molecules and the carbon surface, and consequently improving the adsorption capacity, a finding that is in line with previous studies [51].

The adsorption experiments performed with the catalysts (AC-Fe samples) presented the same tendency as the modified activated carbons. The amount of toluene transferred was very similar—see Figure 4b—because the modified activated carbons and respective catalysts possess similar textural properties (see Table 2).

In summary, the results obtained show that the surface chemical properties played an important role in the adsorption experiments, since the differences in textural properties of all the modified activated carbons were small—Table 2.

**Figure 5:**
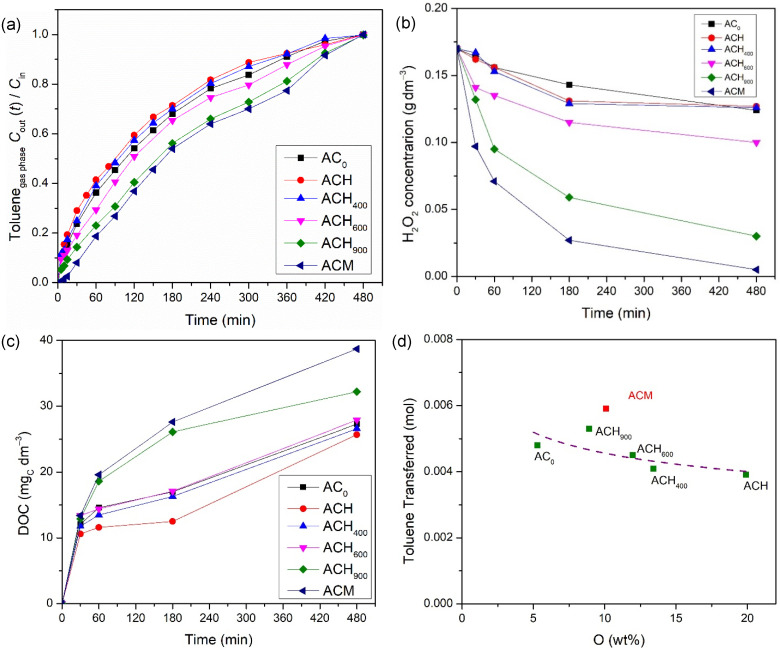
Effect of metal-free catalysts on the toluene concentration at the outlet of the bubble reactor (**a**), on the concentration of H_2_O_2_ (**b**), and on the DOC concentration (**c**) in the liquid phase during wet peroxidation experiments; and correlation between the amount of toluene transferred and the oxygen content present on each modified activated carbon (**d**). Experimental conditions were as follows: pH_0_ = 3.0, [metal-free catalyst] = 0.75 g dm^−3^, [H_2_O_2_] = 0.005 mol dm^−3^, *Q*_air_ = 0.15 dm^3^ min^−1^.

Posteriorly, the catalytic activity of both metal-free catalysts and iron-based catalysts was also evaluated. For that, several runs were carried out under the same operating conditions that were used in the adsorption experiments, but with addition of the oxidant. Figure 5 presents the outlet toluene dimensionless concentration in the gaseous phase; the consumption of H_2_O_2_ during the wet peroxidation experiments, i.e., when using the metal-free catalysts; and the dissolved organic carbon (DOC) in the liquid phase. Regarding Figure 5a, the same tendency obtained in the adsorption with the modified activated carbons was observed, i.e., sample ACH showed the worst performance (overall amount of transferred toluene, η= 3.91 × 10^−3^ mol), while the heat-treated samples reached successively better transfer levels (from 4.10 × 10^−3^ mol to 5.30 × 10^−3^ mol) with increases in the temperature used during the thermal treatment (from 400 °C to 900 °C); this is corroborated by the relationship between the transferred toluene and the amount of oxygen on the carbon-based materials, as demonstrated in Figure 5d. Finally, the highest performance (η= 5.91 × 10^−3^ mol) was obtained with the melamine-doped AC sample (ACM), as reported in other studies but for different applications [24,31]. A reduction in the oxidant concentration was observed in all experiments—Figure 5b—thus corroborating the possible formation of hydroxyl radicals via oxidant consumption, since a clear relationship between the H_2_O_2_ consumption rate and the amount of toluene transferred was observed. Moreover, the dissolved organic carbon (DOC) concentration increased over time—Figure 5c. This increase is related to the formation of intermediate compounds by the oxidation of toluene (and not due to the toluene being absorbed in water, since this compound was not detected on TOC equipment) [25]. The highest DOC concentration was obtained with the ACM sample, reaching 39 mg_C_ dm^−3^, while the lowest DOC concentration recorded was 26 mg_C_ dm^−3^, with the ACH sample; this corresponded to the highest (96.9 %) and lowest (25.2%) consumption of H_2_O_2_, respectively, among the experiments involving the metal-free catalysts. As stated above, these results are in accordance with the performances obtained in terms of the overall amount of toluene transferred in wet peroxidation runs. Higher toluene transfer is associated with a higher consumption of H_2_O_2_. Consequently, a higher amount of H_2_O_2_ consumed implies a higher amount of ${\mathrm{HO}}^{•}$ radicals present in the liquid phase, which degrade the organic compounds that form intermediate compounds, thus increasing the DOC concentration. In summary, the levels reached by adsorption and wet peroxidation with the metal-free catalysts indicate that activated carbons are particularly active for the degradation of toluene via wet peroxidation in a slurry bubble reactor. Obviously, the transfer of toluene from the gas stream and its oxidation generates another problem—a liquid effluent with organic species. As shown in a previous paper [25], this issue can practically be addressed by implementing subsequent oxidation cycles that treat the liquid and the gas; however, this is beyond the scope of this study.

**Figure 6:**
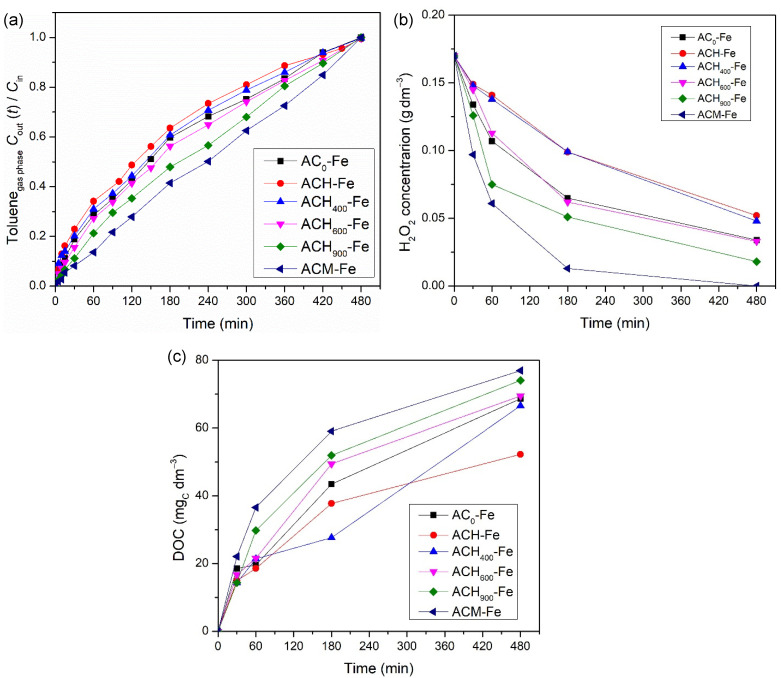
Influence of iron-based catalysts on the toluene concentration at the outlet of the bubble reactor (**a**), on the concentration of H_2_O_2_ (**b**), and on the DOC concentration (**c**) in the liquid phase during heterogeneous Fenton oxidation experiments. Experimental conditions were as follows: pH_0_ = 3.0, [catalyst] = 0.75 g dm^−3^, [H_2_O_2_] = 0.005 mol dm^−3^, *Q*_air_ = 0.15 dm^3^ min^−1^.

In order to determine the influence of the modified activated carbons’ surface chemistry on the catalytic activity of the derived iron-based catalysts, several runs were performed using modified activated carbons impregnated with 2 wt.% of iron in the presence of H_2_O_2_. Figure 6 indicates the activity levels of the different catalysts for toluene treatment by heterogenous Fenton oxidation. The results suggest that the surface chemistry of the modified ACs played an important role in the activity levels reached. Actually, the same tendency that was noticed before with the metal-free catalysts was observed with the iron-based catalysts, and therefore, the sample ACH-Fe presented the worst performance (4.40 × 10^−3^ mol and 52 mg_C_ dm^−3^ in terms of total toluene transferred and final DOC concentration, respectively). The activity level of the heat-treated catalysts was again successively better with increases in the thermal treatment temperature, since the amount of oxygenated surface groups decreased. The best transfer level recorded among all catalysts was by the ACM-Fe sample, reaching 6.39 × 10^−3^ mol of toluene transferred and 77 mg_C_ dm^−3^ as the final DOC concentration, respectively. Moreover, as demonstrated in Figure 6b, the experiment with ACM-Fe consumed all of the hydrogen peroxide present in the liquid phase. As mentioned before, the higher toluene transfer is related to the higher oxidant consumption and, consequently, the higher DOC concentration—see Figure 6c. The presence of nitrogen-containing surface groups increases the amount of π-electron rich sites on the basal planes of the AC materials, favoring adsorption of toluene molecules and their subsequent degradation by Fenton’s reagents. Moreover, the nitrogen-containing surface groups can maintain the iron species near the carbon surface, enabling the hydrogen peroxide to more easily reach the iron species that favors the formation of hydroxyl radicals [52].

**Figure 7:**
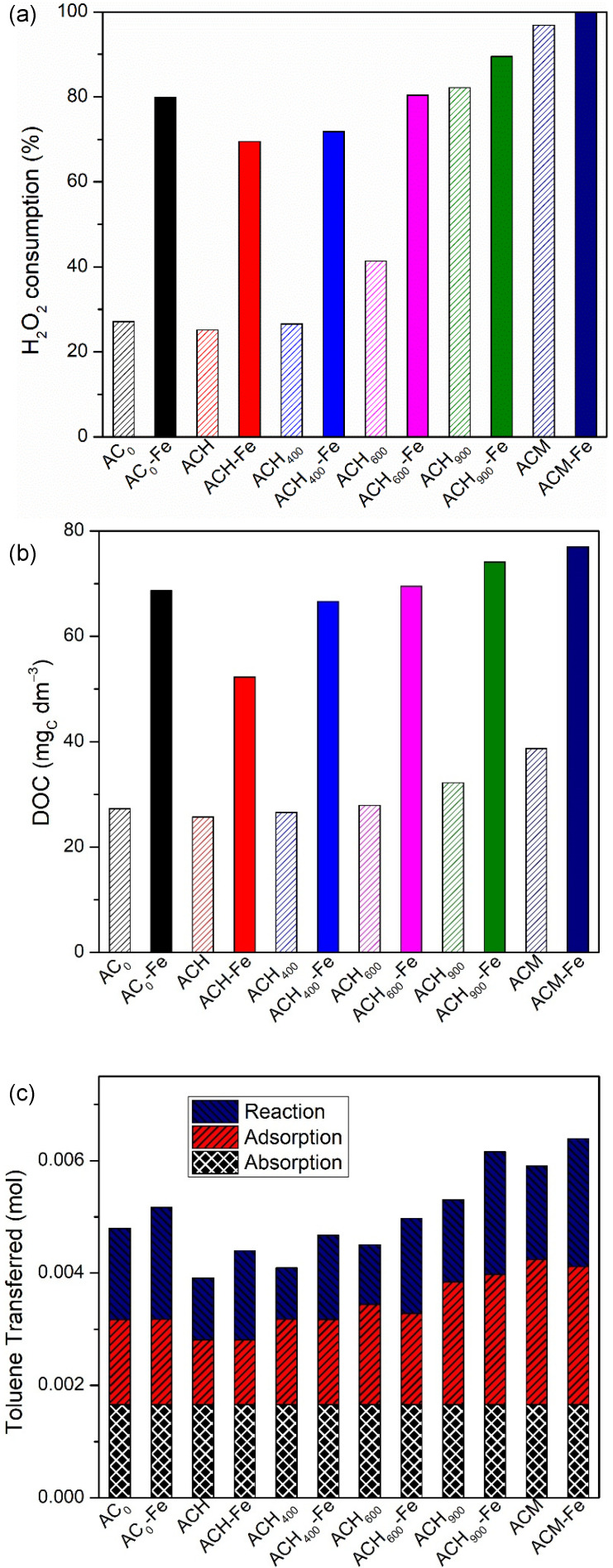
H_2_O_2_ consumption (%) (**a**), final DOC concentration (mg_C_ dm^−3^) (**b**) and the contribution of each process (**c**) for all metal-free catalysts and iron-based catalysts used for the degradation of gaseous toluene in the bubble reactor. Experimental conditions were as follows: pH_0_ = 3.0, [metal-free catalyst/iron-based catalyst] = 0.75 g dm^−3^, [H_2_O_2_] = 0.005 mol dm^−3^, *Q*_air_ = 0.15 dm^3^ min^−1^, *t* _reaction_ = 480 min.

Figure 7a shows the overall H_2_O_2_ consumption during the catalytic oxidation (wet peroxidation and heterogeneous Fenton oxidation) for all carbon materials prepared. As mentioned above, the highest consumption was recorded by the ACM sample and its respective catalyst, while the lowest consumption was obtained by ACH. Furthermore, the consumption of oxidant in wet peroxidation (with the metal-free catalysts) was lower than the consumption in the heterogeneous Fenton reaction (with the corresponding iron-based catalysts), because of the higher activity of Fe-based catalysts compared to the corresponding activated carbon. Inherently, the DOC generated was higher for the AC-Fe catalysts. In fact, from Figure 7b one can see that the final DOC concentration with the Fe-catalysts is approximately twice as much the final DOC concentration with the metal-free catalysts; for example, the values of 77 mg_C_ dm^−3^ and 39 mg_C_ dm^−3^ were associated with the ACM-Fe and ACM samples, respectively. Figure 7c presents the overall toluene transferred, and the contribution of each process (absorption, adsorption and catalytic oxidation) for all carbon materials used. Among the metal-free catalysts and Fe-catalysts, the best performances observed were by the sample doped with nitrogen, i.e., ACM and ACM-Fe, respectively, for the reasons discussed above. Both samples showed a similar consumption of hydrogen peroxide (97% and 100%, respectively—Figure 7a). The differences between them (as well as between any modified activated carbon and the corresponding iron-based catalyst) are explained on the basis of the pathways for hydroxyl radicals generation: (i) reaction between the modified activated carbon with H_2_O_2_ (Equation (8)) [53] and (ii) catalytic decomposition of the H_2_O_2_ molecules in the presence of iron (Equation (5)) [19], where X represents the modified activated carbon.

(9) $${X+H}_{2}O_{2\;}\to {\;X}^{+}{+\mathrm{HO}}^{•}+\mathrm{OH}$$

As expected, and as shown in Figure 7c, the absorption contribution is the same for all materials, but the contribution of adsorption and reaction varies from sample to sample. In all pairs of modified activated carbon and respective iron-based catalyst, the adsorption contribution for toluene capture was similar due to the similar textural properties, but the one of reaction is always higher for the Fe-catalyst, for the reasons discussed before.

**Figure 8:**
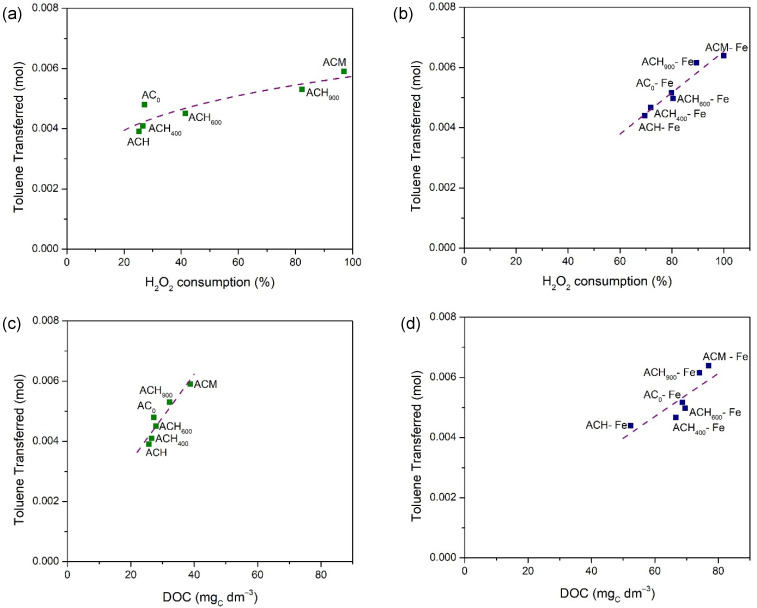
Correlations between the amount of toluene transferred and H_2_O_2_ consumption for metal-free activated carbons (**a**) and for Fe-derived catalysts (**b**); and final DOC concentrations for metal-free activated carbons (**c**) and for Fe-derived catalysts (**d**).

The results discussed above show the existence of a correlation between the amount of toluene transferred and the H_2_O_2_ consumption or final DOC concentration, as shown Figure 8, either for the metal-free activated carbons (Figure 8a,c) or the iron-based catalysts (Figure 8b,d).

In a previous study, Rodrigues et al. [49] assessed the toluene degradation in a bubble column reactor (BCR) catalyzed by a carbon-coated monolith that was impregnated with iron (CM-Fe), and the maximum amount transferred was 1 × 10^−2^ mol of toluene for the same volume of water (300 mL), but with different doses of catalyst (2.3 g L^−1^ vs. 0.75 g L^−1^ employed in the present study). However, a comparison between these studies is challenging because of the different catalyst doses and reactor configurations involved.

One important issue of the industrial use of the Fe-based catalysts is their stability, and particularly the level of metal leaching. Table 5 shows the amount of iron leaching during heterogeneous Fenton oxidation with all AC-Fe samples. The iron concentration in solution is always significantly below the legislated standard of 2 mg_Fe_ dm^−3^ [54], corresponding to a leaching level < 0.6% of the iron that was initially present in the catalysts.

#### 3.2.3. Stability of the ACM-Activated Carbon and ACM-Fe Catalyst

**Figure 9:**
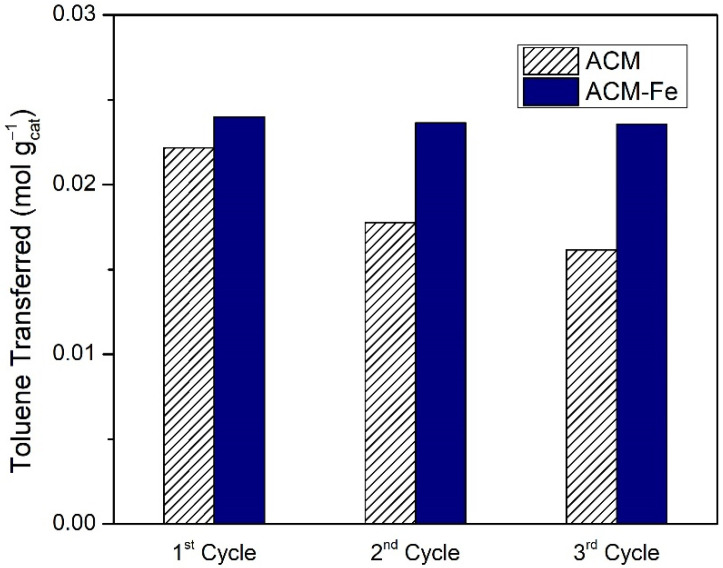
Toluene transferred per mass of modified activated carbon/iron-based catalyst used in subsequent cycles during wet peroxidation with the ACM sample and Fenton’s reaction with ACM-Fe catalyst. Experimental conditions were as follows: pH_0_ = 3.0, [H_2_O_2_] = 0.005 mol L^−1^, *Q*_air_ = 0.15 dm^3^ min^−1^.

As seen in the previous section, the best catalytic oxidation of toluene was obtained in the presence of the iron-based catalyst doped with nitrogen (ACM-Fe sample) and the respective activated carbon (ACM sample), resulting in similar amounts of toluene transferred, namely 6.39 × 10^−3^ mol and 5.91 × 10^−3^ mol, respectively. The question that remains is whether it is worth impregnating the ACM sample with Fe or using the modified activated carbon directly. The differences among them may be found through investigating their stability in consecutive reaction cycles under the same operating conditions. The samples after each cycle were recovered using filtration, and were used again after drying at room temperature. However, during the filtration process there is some loss of catalyst mass between cycles. Therefore, Figure 9 presents the toluene transferred per mass of carbon material used in each of the consecutive cycles. The catalytic performance of the ACM sample decreased successively with each cycle, probably as a result of the porosity saturation along the cycles (BET surface area of the ACM decreased from 807 m^2^ g^−1^ to *S*_BET_ = 525 m^2^ g^−1^ after one cycle and *S*_BET_ = 275 m^2^ g^−1^ after three cycles) and/or deactivation of the carbon material from oxidation of the carbon surface by the action of H_2_O_2_, which provokes the formation of oxygen surface groups [24,55,56]. On the other hand, the catalytic performance of ACM-Fe was essentially maintained during the three cycles, although a slight deactivation was observed that was in line with the low cumulative leach of iron during the reutilization cycles (Table 5).

In conclusion, the results show that the use of an iron-based catalyst doped with nitrogen resulted in the best overall performance, which was quite stable across three reutilization cycles, thus making it advantageous for real applications. However, many more cycles should be conducted in order to prove its long-term stability.

## 4. Conclusions

To the best of the authors’ knowledge, modified activated carbons and iron-based catalysts were used for the first time in toluene gas stream degradation by heterogeneous Fenton oxidation in a bubble reactor. It was found that their surface chemical properties play a key role in adsorption and/or catalytic activity. The worst performances for adsorption, wet peroxidation, and Fenton oxidation occurred with the ACH and ACH-Fe samples, which presented the highest amount of oxygen groups on the material surface. The performance of AC-based materials increased successively with the removal of more of these oxygen surface groups through thermal treatment at different temperatures. However, the best performance among all tested samples was obtained in the presence of the activated carbon doped with nitrogen and impregnated with iron—the ACM-Fe sample (yielding 6.39 × 10^−3^ mol of toluene transferred)—highlighting the important role that nitrogen-containing groups play in the catalytic performance of the synthesized materials. Moreover, the ACM-Fe sample was found to be quite stable over three reutilization cycles.

Heterogeneous Fenton oxidation proved to be a promising technology for toluene degradation in gas streams, particularly when using an ACM-Fe iron-based catalyst in a slurry bubble reactor.

## Figures and Tables

**Table 1:** Abbreviations of the samples and descriptions of the treatments applied.

Acronym	Initial Material	Description of Treatment
AC_0_	Original	None
ACH	AC_0_	Oxidation in liquid phase with 6 M of HNO_3_
ACH_400_	ACH	Thermal treatment under N_2_ flow at 400 °C
ACH_600_	ACH	Thermal treatment under N_2_ flow at 600 °C
ACH_900_	ACH	Thermal treatment under N_2_ flow at 900 °C
ACM	AC_0_	N-doping using melamine as nitrogen source
ACX_Y_-Fe	Altered activated carbons	Incipient wetness impregnation method

X_Y_—symbolizes the modification that occurred on the different samples.

**Table 2:** Textural properties of the modified activated carbons and their respective iron-based catalysts.

Material	*S*_BET_ (±20 m^2^g^−1^)	*S*_meso_ (±20 m^2^g^−1^)	*V*_micro _ (±0.01 cm^3^g^−1^)	*V*_p _ (±0.01 cm^3^g^−1^)
AC_0_	1047	170	0.36	0.54
ACH	968	151	0.35	0.52
ACH_400_	1011	159	0.32	0.49
ACH_600_	1072	182	0.38	0.58
ACH_900_	1059	160	0.39	0.56
ACM	807	125	0.28	0.42
AC_0_-Fe	983	162	0.34	0.53
ACH-Fe	1010	173	0.35	0.55
ACH_400_-Fe	941	165	0.33	0.50
ACH_600_-Fe	1043	181	0.36	0.58
ACH_900_-Fe	1015	171	0.35	0.53
ACM-Fe	823	84	0.32	0.44

**Table 3:** Elemental analysis and pH_PZC_ values of the modified activated carbons.

Sample	C (wt.%)	H (wt.%)	N (wt.%)	S (wt.%)	O (wt.%)	pH_PZC_
AC_0_	78.60	1.94	0.00	0.08	5.28	7.6
ACH	71.50	2.84	0.63	0.03	19.91	2.6
ACH_400_	72.80	2.48	0.01	0.02	13.42	6.0
ACH_600_	76.80	2.64	0.02	0.04	11.97	7.3
ACH_900_	81.10	1.99	0.00	0.14	8.92	7.5
ACM	76.30	2.64	7.01	0.06	10.08	7.5

Other elements contained in the activated carbons (namely inorganic) were not determined through the elemental analysis.

**Table 4:** Proximate analysis of the modified activated carbons.

Material	Weight (%)		
	Volatile Content	Fixed Carbon	Ash
AC_0_	8	75	17
ACH	35	57	8
ACH_400_	16	69	15
ACH_600_	13	68	19
ACH_900_	8	80	12
ACM	30	47	23

**Table 5:** Iron content and amount of iron leached in heterogeneous Fenton oxidation with different catalysts.

Material	Iron Content (%)	Leached Iron (mg_Fe_ dm^−3^)	Leached Iron (%)
AC_0_-Fe	2.2	0.046	0.31
ACH-Fe	2.1	0.054	0.36
ACH_400_-Fe	2.0	0.080	0.53
ACH_600_-Fe	1.8	0.070	0.47
ACH_900_-Fe	1.7	0.040	0.27
ACM-Fe (1st cycle)	1.8	0.037	0.25
ACM-Fe (2nd cycle)	-	0.005	0.41
ACM-Fe (3rd cycle)	-	0.022	0.21

## Supplementary Materials

The following supporting information can be downloaded at: https://www.mdpi.com/article/10.3390/nano12193274/s1. Figure S1. N_2_ adsorption isotherms (a,b) and pore size distribution (c,d) of the modified activated carbons (a,c) and respective iron-containing catalysts (b,d). Figure S2. Thermogravimetric profiles of the modified activated carbons. Figure S3. Particle size distribution of ACM-Fe fresh sample (a), ACM-Fe used sample (b), AC_0_-Fe fresh sample (c), ACH-Fe fresh sample (d) and ACH_900_-Fe fresh sample (e).
