# Supplementary material for: Degradation of Toluene from Gas Streams by Heterogeneous Fenton Oxidation in a Slurry Bubble Reactor with Activated Carbon-Based Catalysts
Source: Nanomaterials (Basel). 2022 Sep 21;12(19):3274. doi: 10.3390/nano12193274 (PMC9565738; doi:10.3390/nano12193274)
Supplement: Supplementary file 1 [file nanomaterials-12-03274-s001.zip › nanomaterials-1871098-supplementary.pdf]

Supporting Information

Degradation of Toluene from Gas Streams by Heterogeneous Fenton Oxidation in a Slurry Bubble Reactor with Activated Carbon-Based Catalysts

Emanuel F. S. Sampaio [1,2,3], V. Guimarães [1,2,3], O. S. G. P. Soares [2,3], M. Fernando R. Pereira [2,3], Carmen S. D. Rodrigues [1,3] and Luis M. Madeira [1,3,*]

[1] LEPABE—Laboratory for Process Engineering, Environment, Biotechnology and Energy, Faculty of Engineering, University of Porto, Rua Dr. Roberto Frias, 4200-465 Porto, Portugal

[2] LSRE–LCM—Laboratory of Separation and Reaction Engineering—Laboratory of Catalysis and Materials, Faculty of Engineering, University of Porto, Rua Dr. Roberto Frias, 4200-465 Porto, Portugal

[3] ALiCE—Associated Laboratory in Chemical Engineering, Faculty of Engineering, University of Porto, Rua Dr. Roberto Frias, 4200-465 Porto, Portugal

* Correspondence: mmadeira@fe.up.pt ; Tel.: + 351-22-508-1519; Fax: +351-22-508-1449

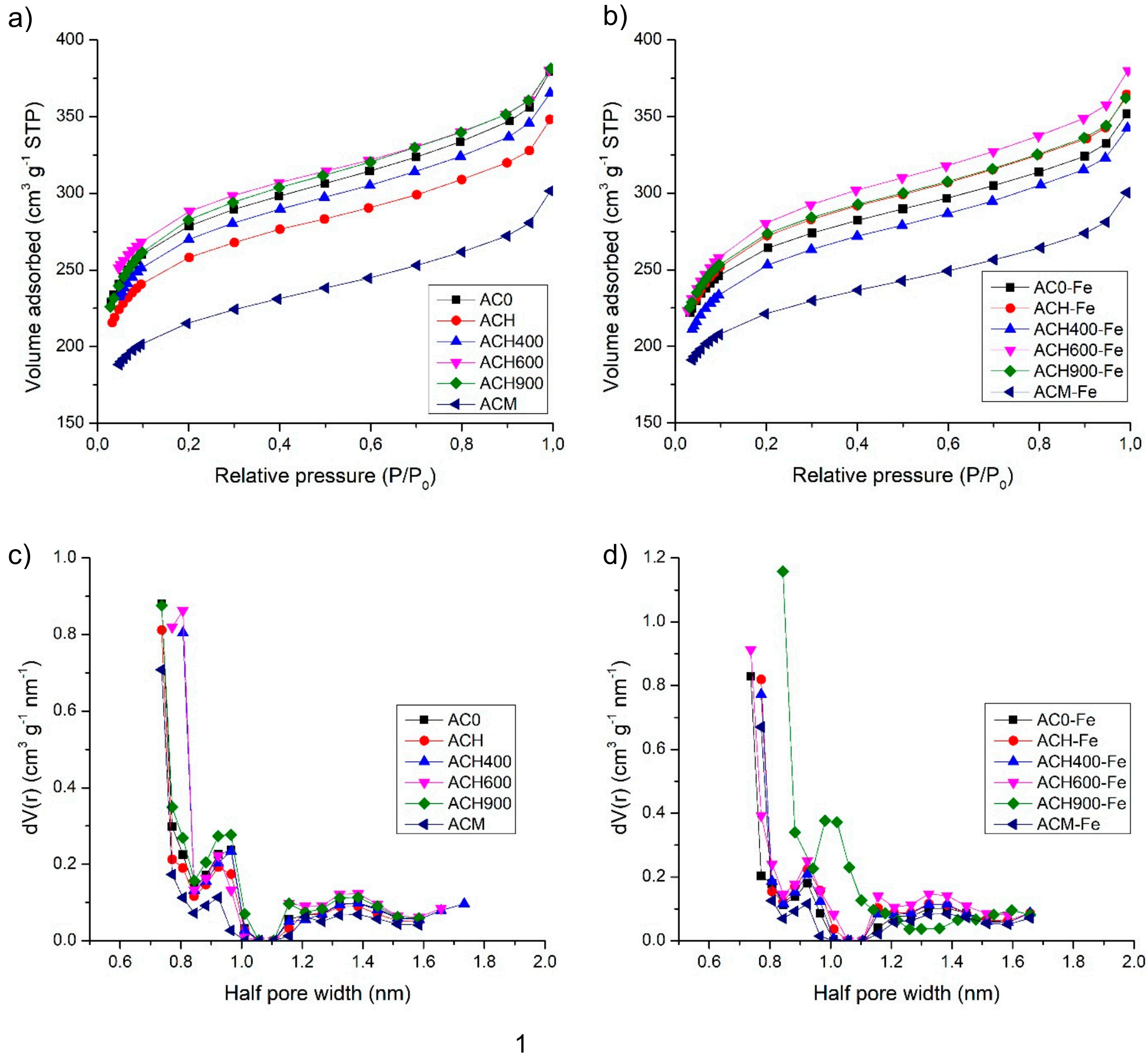

Figure S1 - N_2 adsorption isotherms (**a**,**b**) and pore size distribution (**c**,**d**) of the modified activated carbons (**a**,**c**) and respective iron-containing catalysts (**b**,**d**).

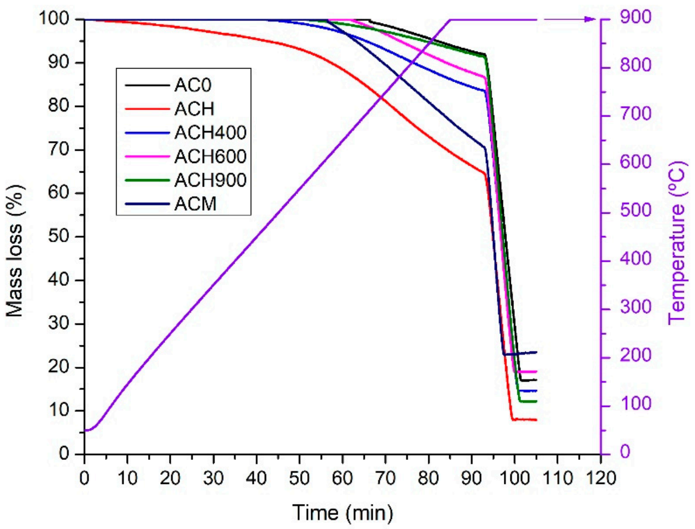

Figure S2 - Thermogravimetric profiles of the modified activated carbons.

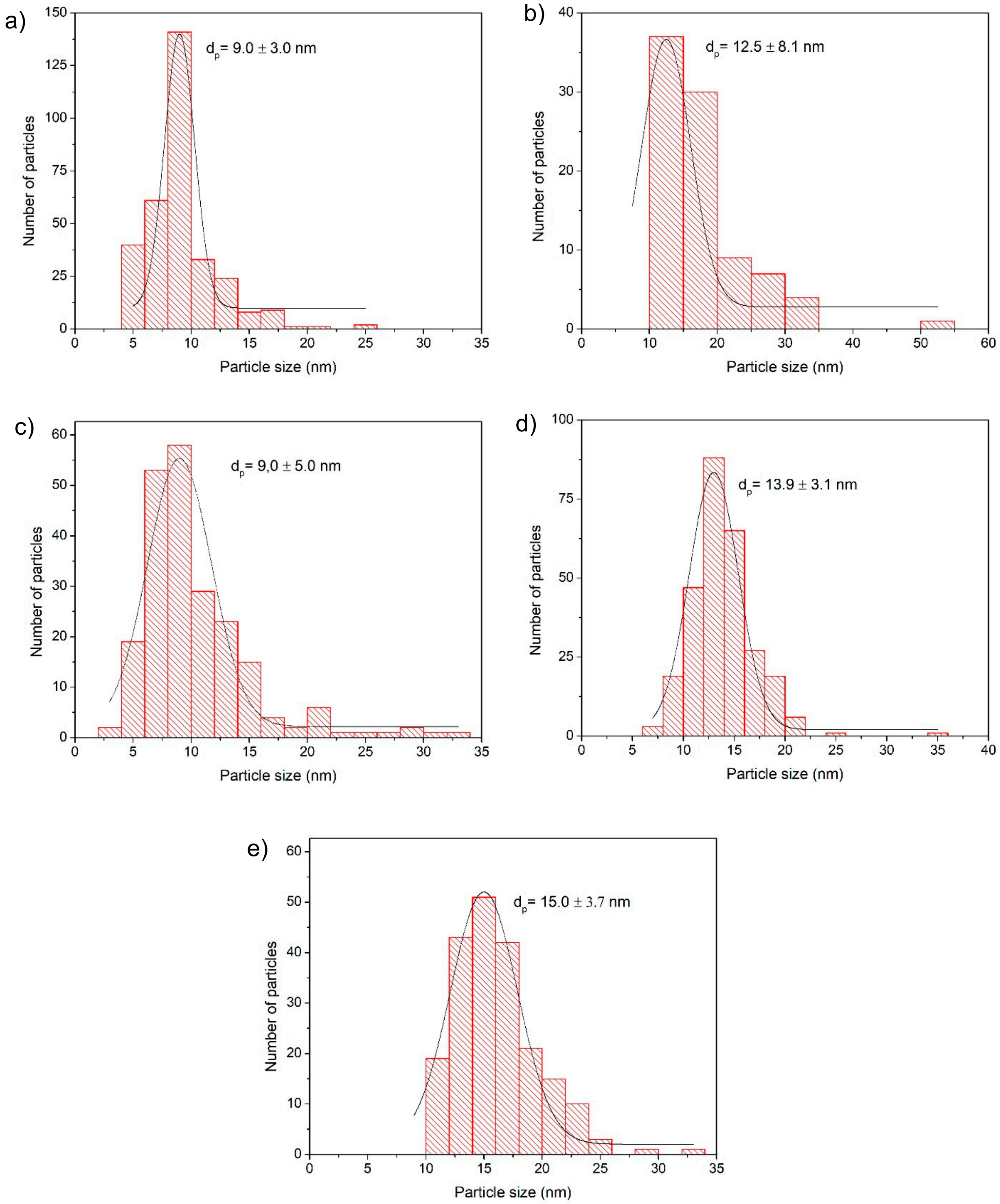

Figure S3 – Particle size distribution of ACM-Fe fresh sample (**a**), ACM-Fe used sample (**b**), AC_0-Fe fresh sample (**c**), ACH-Fe fresh sample (**d**) and ACH_{900}-Fe fresh sample (**e**).
